# In patients with suspected thrombotic thrombocytopenic purpura, what is the optimal time to therapeutic plasma exchange?

**DOI:** 10.1016/j.htct.2025.106223

**Published:** 2025-12-12

**Authors:** Alexandre Soares Ferreira Junior, Kate Sanborn, Morgana Pinheiro Maux Lessa, Alexander Gordee, Maragatha Kuchibhatla, Allison O. Taylor, Matthew S. Karafin, Oluwatoyosi A. Onwuemene

**Affiliations:** a Department of Medicine, Faculdade de Medicina de São José do Rio Preto, São José do Rio Preto, São Paulo, Brazil; b General and Applied Biology Program, Institute of Biosciences, Sao Paulo State University, Botucatu, Brazil; c Duke Biostatistics, Epidemiology and Research Design Core, Duke University School of Medicine, Durham, NC, USA; d Department of Biostatistics and Bioinformatics, Duke University School of Medicine, Durham, NC, USA; e Division of Hematology, Department of Medicine, Duke University School of Medicine, Durham, NC, USA; f Department of Pathology and Laboratory Medicine, University of NC, Chapel Hill, NC, USA

**Keywords:** ADAMTS13, Hemostasis, Plasmapheresis, Thrombotic thrombocytopenic purpura, Thrombocytopenia

## Abstract

**Background:**

In patients with suspected immune thrombotic thrombocytopenic purpura, guidelines suggest that therapeutic plasma exchange should be initiated within eight hours. However, this time threshold may be difficult to attain. This study sought to identify the optimal time to plasma exchange to maximize outcomes.

**Study Design and Methods:**

Patients with international classification of disease codes for thrombotic microangiopathy were identified in a retrospective cross-sectional analysis of public use data files from the Recipient Epidemiology and Donor Evaluation Study-III (REDS-III). The assumption of linearity between time to therapeutic plasma exchange and the composite outcome of bleeding, thrombosis, and mortality were evaluated. Subsequently, the optimal time for plasma exchange was identified using a nonparametric approach with bootstrapping.

**Results:**

For 149 patients with a suspected diagnosis of thrombotic thrombocytopenic purpura, the association between time to plasma exchange and the primary outcome was non-linear. With regard to the primary composite outcome, this time had a low predictive capacity (area under the curve: 0.62). The optimal time that maximized outcomes was 13.5 h.

**Conclusion:**

Although this study found that time to therapeutic plasma exchange did not independently predict outcome, future studies might evaluate how this time interacts with other variables to predict clinical outcomes.

## Introduction

In patients with suspected thrombotic thrombocytopenic purpura (TTP), therapeutic plasma exchange (TPE) and immunosuppression should be initiated promptly [1]. Although prompt TPE initiation has been associated with improved outcomes of thrombosis, major bleeding, and mortality, the optimal time to TPE that also minimizes poor outcomes is unknown [2–4]. Based on guidelines from the British Society of Haematology, the recommended time window between referral for a suspected diagnosis and TPE initiation is 4–8 h [1]. However, this specific time window may lack robust supporting evidence. Also, due to barriers in diagnosis and treatment initiation, this time window is often not attained [3,5].

Prior evidence suggests that in patients with suspected TTP, TPE initiation within 8 h is achieved in only 27–41 % of patients [3,5]. In fact, time to TPE has been found to be delayed as long as >72 h [4,6]. In studies evaluating treatment delays >24 to 48 h, delays longer than 8 h may account for differences in thrombosis, major bleeding, and mortality outcomes [2,4]. Therefore, there is a need to identify the optimal time window within which TPE initiation is both practicable and associated with improved outcomes. Identifying an optimal TPE initiation window may both simplify clinical practice and guide timely diagnostic strategies.

To characterize the association between time to TPE and outcomes and identify the optimal time threshold to TPE initiation, this study analyzed public use data from the Recipient Epidemiology and Donor Evaluation Study-III (REDS-III) [7,8].

## Methods

### Study design and data source

This study is a retrospective cross-sectional analysis of public use data files from the REDS-III study [9]. REDS-III was a prospective longitudinal four-year (2013–2016) National Heart, Lung and Blood Institute-sponsored observational multicenter electronic health record study involving four United States blood centers and 12 hospitals [7,8]. Participating sites contributed data on blood donors/donations (6.5 million blood components) and transfusion recipients (120,290 patients in over 234,277 encounters) [7,9,10]. Included as a comparison group were all non-transfused patients in inpatient encounters (1,285,359). REDS-III data include demographics, laboratory results, medication administration, and blood product transfusions [11]. Because REDS-III public use data files contain de-identified data, the study was reviewed by the Institutional Review Board and determined to be exempt.

### Participant selection

All children and adults were included in this study if they had a suspected TTP diagnosis. To identify patients with suspected TTP, a validated strategy was used that has been identified to have a positive predictive value of 65 % [12]. Thus, participants with suspected TTP were identified using common procedural terminology (CPT), and ICD-9 and 10 (International Classification of Diseases, ninth and tenth edition) codes for TPE (CPT code 36514, or ICD-9 code 99.71, or ICD-10 codes 6A550Z3 or 6A551Z3) and thrombotic microangiopathy (TMA: ICD-9 446.6 or ICD-10 M31.1; see codes in the Appendix) [2,12,13]. Although REDS-III provides data at the encounter level, this analysis was done at a patient level. Patient level data were obtained by merging recipient data files by encounter and subject ID [11]. Additionally, because each patient may have multiple healthcare encounters, this analysis included only data from the first suspected TTP inpatient encounter (unique hospitalization event).

### Primary outcome and variable definitions

The primary outcome was defined as a composite of arterial and venous thrombosis, major bleeding and all-cause mortality (see ICD code definitions in the Appendix) [2,4]. Time to TPE was defined as the time from the recorded hospital admission to the first plasma issue time reported in hours [2,5]. Time to platelet recovery was defined as days from first plasma issue to platelet count >150 × 10^9^/L [14]. Refractory TTP was defined as no platelet count doubling and lactate dehydrogenase concentration greater than the upper limit of the normal range after four days of treatment [14]. TTP exacerbation was defined as a subsequent TTP encounter occurring <30 days after discharge [15]. TTP relapse was defined as a subsequent TTP encounter occurring ≥30 days after discharge [15]. Plasma issued from the blood bank was identified using validated Information Standard for Blood and Transplant (ISBT) codes [8,10,11].

## Statistical analysis

To determine the optimal time to TPE, this study sought to identify the time threshold that maximized Youden’s statistic (sensitivity + specificity – 1) – the cutoff-point that provides the best balance between true positive and true negative rates [16]. To this end, the assumption of linearity between time to TPE and log odds of our primary outcome was evaluated.

To determine the optimal time threshold, a nonparametric approach was used with the R (version 4.4.0) “cutpointr” package [17]. The “cutpointr” package allows direct estimation of cutoff values in binary classification problems. To this end, various metrics were calculated for a range of potential time cutoff values [17]. Thus, each possible time threshold was evaluated by 1) calculating its sensitivity and specificity, 2) computing Youden’s index, and 3) selecting the time threshold that maximizes Youden’s index.

To optimize the robustness of the identified time threshold, bootstrapping (1000 resamples) was performed. By repeatedly resampling and replacing the dataset and recalculating the optimal threshold, bootstrapping enhances the reliability of the identified time threshold across multiple samples.

### Sensitivity analysis

#### Clustering on time to TPE

In the course of the analysis, a non-linear association was identified between time to TPE and the odds of the primary outcome. Therefore, clusters, based on time to TPE, were investigated to identify subpopulations within the cohort. The aligned box criterion was used to select the optimal number of clusters. This criterion helps identify the optimal number of clusters (k) that are not only compact and distinct but also aligned with the underlying data [18]. This analysis was performed using k-means clustering within PROC HPCLUS in SAS version 9.4 (SAS Institute Inc., Cary, NC). These clusters were used in the sensitivity analysis below.

#### Comparison of multiple models and datasets

To increase the specificity of the TTP cohort and validate the consistency of the findings, a series of sensitivity analyses was performed. First, time to TPE (≥5 days) was used as an exposure variable to identify outliers. Second, outliers based on the cluster analysis were excluded; and third, cases that received only one TPE procedure (ICD 10 codes distinguish only single versus multiple procedures) were excluded [19,20]. Different parametric and nonparametric tests were performed for each sub-analysis. Each test was done with and without bootstrapping (see Supplementary Methods).

## Results

### Baseline characteristics and outcomes

From 2012 to 2016, the number of patients meeting inclusion criteria for a suspected TTP diagnosis was 149. Among these, the mean age in years was 50. Most patients were female (70.5 %), White (64.4 %), and non-Hispanic (88.6 %). Average admission lab values (± standard deviation [SD]) were as follows: 1) hemoglobin (9.7 ± 2.3 g/dL); 2) platelet count (69.8 ± 85.9 × 10^9^); 3) lactate dehydrogenase (1056.8 ± 898.1 U/L); and 4) creatinine (2.1 ± 1.8 mg/dL). The average time to TPE was 71.3 ± 136.4 h. Detailed baseline characteristics and demographic information are shown in Table 1.

**Table 1:** Baseline characteristics of patients with suspected thrombotic thrombocytopenic purpura.

	**Suspected TTP (*n*=149)**
**Demographic**	
**Age**	
Mean (SD)	50.2 (18.2)
Median	50.0
Range	(7.0–87.0)
**Gender – n (%)**	
Male	44 (29.5)
Female	105 (70.5)
**Race – n (%)**	
White	96 (64.4)
Black or African American	33 (22.1)
Asian	3 (2.0)
Other	7 (4.7)
Not Reported	10 (6.7)
**Ethnicity – n (%)**	
Hispanic	7 (4.7)
Non-Hispanic	132 (88.6)
Not Specified	10 (6.7)
**Comorbidity – n (%)**	
Diabetes	34 (22.8)
Heart Failure	18 (12.1)
Renal Disease	99 (66.4)
Hepatic Disease	17 (11.4)
Stroke	19 (12.8)
Transient Ischemic Attack	2 (1.3)
Venous Thrombosis	26 (17.4)
Pulmonary Embolism	8 (5.4)
Immune Thrombocytopenia	11 (7.4)
Evans Syndrome	1 (0.7)
SLE	6 (4.0)
APS	12 (8.1)
**Admission Labs**	
**Hemoglobin (g/dL)**	
n	149
Mean (SD)	9.7 (2.3)
Median	9.4
Range	(4.7–16.4)
**Platelet count (x 10^9^)**	
n	148
Mean (SD)	69.8 (85.9)
Median	36.5
Range	(4.0–464.0)
**Lactate Dehydrogenase (U/L)**	
n	148
Mean (SD)	1056.8 (898.1)
Median	729.0
Range	(126.0–5002.0)
**Creatinine (mg/dL)**	
n	149
Mean (SD)	2.1 (1.8)
Median	1.3
Range	(0.5–14.2)
**Troponin (ng/mL)**	
n	53
Mean (SD)	2.6 (13.1)
Median	0.1
Range	(0.0–96.0)
**Time to TPE (h)**	
Mean (SD)	71.3 (136.4)
Median	23.5
Range	(0.6–1179.6)

TTP: thrombotic thrombocytopenic purpura; APS: Antiphospholipid syndrome; SD: Standard deviation; SLE: Systemic Lupus Erythematosus; TTP: Thrombotic thrombocytopenic purpura.

### Association between time to therapeutic plasma exchange and the primary outcome

Outcomes data are shown in Table 2.

**Table 2:** Outcomes of 149 patients with suspected thrombotic thrombocytopenic purpura.

	**Suspected TTP**
**Composite outcome n (%)**	90 (60.4)
**Time to platelet recovery** (days)	
Mean (SD)	7.6 (8.0)
Median	4.6
Range	(0.3–39.5)
Refractory TTP n (%)*	25 (28.1)
**TTP relapse n (%)**	8 (5.4)
**TTP exacerbation n (%)**	19 (12.8)

SD: Standard deviation; TPE: Therapeutic plasma exchange; TTP: Thrombotic thrombocytopenic purpura.

⁎ Included data from 89 patients.

**Figure 1:**
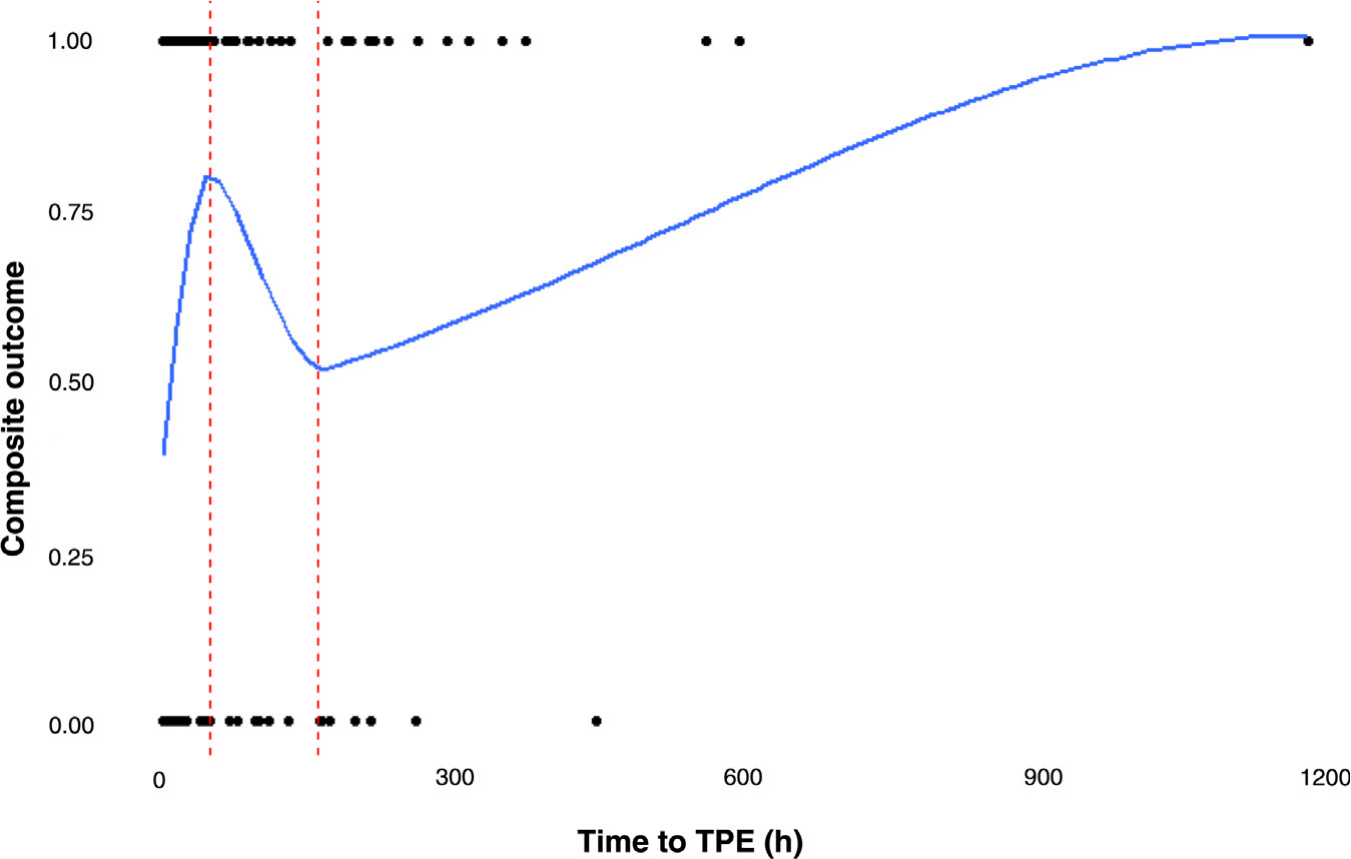
The association between time to therapeutic plasma exchange and log odds of composite outcome is non-linear. TPE: Therapeutic plasma exchange; TTP: Thrombotic thrombocytopenic purpura.

The association between time to TPE and the log odds of the primary outcome was non-linear (see Figure 1). In the nonparametric model, the optimal time threshold for TPE initiation was 13.5 h (also see bootstrapping analyses in Supplementary Results); and the area under the curve (AUC) was 0.62.

### Sensitivity analysis

The cluster analysis based on time to TPE identified six subpopulations. These subpopulations had distinct baseline characteristics (age and sex), admission labs (platelet count, hemoglobin, creatinine and lactate dehydrogenase levels) and prognosis. In summary, some subpopulations had worse outcomes despite shorter time to TPE initiation (see detailed clustering analysis data in Supplementary Results).

The sensitivity analysis identified a similar non-linear association between time to TPE and the primary composite outcome. Additionally, it identified similar AUCs (range: 0.59–0.66) and time thresholds for TPE initiation (range: 12.98–15.93 h; see Table 3).

**Table 3:** Sensitivity analysis to identify the optimal time threshold for therapeutic plasma exchange initiation.

**Cohort (n)**	**Model**	**AUC**	**Threshold identified (h)**
Total cohort (149)	Parametric	0.65	15.93
Remove those with only one TPE (65)		0.59	13.55
Remove time to TPE >5 days (125)		0.65	13.5
Remove clusters with outliers (139)		0.66	12.98
Remove clusters with 100–200 h (137)		0.66	13.69
Total cohort (149)	Nonparametric (GAM)	0.65	15.93
Removing 100–200 h cluster (137)	Nonparametric with bootstrapping*	0.64	13.5

AUC: Area under the curve; GAM: Generalized Additive Modeling; TPE: Therapeutic plasma exchange;.

⁎ : “cutpointr” package in R.

## Discussion

In this retrospective analysis of patients with suspected TTP, the optimal TPE initiation time threshold was identified as 13.5 h. Additionally, the association between time to TPE initiation and the primary composite outcome (bleeding, thrombosis, and mortality) was found to be non-linear. With regard to the composite outcome, time to TPE initiation had an AUC of 0.62 (poor predictive capacity). Taken together, these data suggest that, although there is a clearly identified threshold, time to TPE initiation may be only one of several factors impacting outcomes.

An important and novel finding of this analysis was the optimal TPE initiation time threshold of 13.5 h. With the sensitivity analyses taking into account outliers and number of TPE procedures, the optimal time threshold may fall between 12.98–15.93 h (see Table 3). To our knowledge, the optimal time to TPE has not been specifically investigated in previous studies. However, compared to the evaluation of time to TPE initiation as a continuous variable in this study, previous studies have evaluated differences in outcomes based on pre-specified time thresholds (that is time to TPE initiation as a categorical variable). In a study of 61 patients with confirmed TTP, outcomes did not differ significantly when the selected categorical time threshold was 8 h [3]. Specifically, when compared to >8 h, TPE initiation within 8 h did not significantly improve rates of myocardial infarction (31 % versus 24 %) neurological events (28 % versus 33 %), venous thromboembolism (6 % versus 10 %) and mortality (7 % versus 4 %). In a previous large database study of 793 cases of suspected TTP, when categorical time thresholds of <1 day, 1 day, 2 days, and >2 days were evaluated, a higher odds of mortality, major bleeding, and thrombosis was associated with time to TPE initiation >2 days (OR: 1.68; 95 % confidence interval: 1.11–2.54; p-value = 0.0150) [2]. Although these studies differ from the current study in their use of time to TPE as a categorical variable, they appear to suggest that the optimal time to TPE that maximizes outcomes is greater than 8 h but <48 h [2,3]. While imperfect (a definitive TTP diagnosis was not established), the methodology used in this study may offer practical insights for improving patient outcomes through timely intervention.

**Figure 2:**
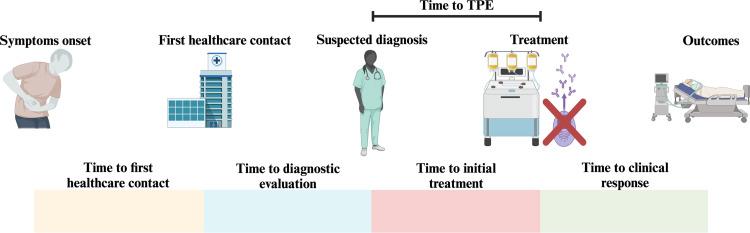
The patient journey since symptoms onset and potential factors contributing to time to diagnosis. TPE: Therapeutic plasma exchange; TTP: Thrombotic thrombocytopenic purpura.

Notwithstanding the above, the ability to compare outcomes across studies is limited by the absence of a definition for standardized time to TPE. In this study, time to TPE was defined as hours from hospital admission to first plasma issue. In other studies, time to TPE has been variably defined as follows: 1) days from hospital admission to first TPE procedure [2,4,13]; 2) hours from laboratory blood sample receipt to blood bank plasma release [5]; and 3) hours from suspected diagnosis to first TPE procedure [3]. Each of these definitions, which serve as an imperfect surrogate for time from suspected diagnosis to TPE initiation, has its own limitations [1]. For example, time to plasma issue could represent, not plasma for TPE but rather, plasma for infusion as a temporizing strategy prior to initiating TPE. Additionally, it may not account for time from blood bank issue to the actual TPE procedure start time. Therefore, in evaluating the impact of time to TPE on TTP outcomes, differences in time definition may confound the results. Furthermore, when the total time from symptoms onset to initial healthcare-seeking behavior and final suspicion is considered, healthcare-associated measures of time to TPE may represent only a small fraction (see Figure 2) [3,6]. In a previous study of 38 patients, the median time from symptoms onset to first hospital visit was 6.5 days (range: 2–18 days) [6]. Thus, measures of time to TPE used here are imprecise: They do not account for delays in healthcare seeking and time from initial healthcare presentation to final acute care referral.

An interesting finding from this analysis is that, with regard to the primary composite outcome, time to TPE had low predictive capacity (AUC: 0.62). While this AUC falls within the range of an “acceptable” predictor, it is lower than what is seen in other commonly used prediction models such as the PLASMIC score (AUC: 0.91–0.96) [21,22]. However, it is important to note that, similar to the PLASMIC score, most prediction models include not one but a combination of several predictive factors [23,24]. Indeed, in patients with TTP, factors known to impact outcomes include age [24, 25, 26], renal failure [24,25], lactate dehydrogenase levels [24,26], platelet count [24], stupor or coma [24,26], and platelet transfusions [25]. This study did not assess the interaction of these variables with time. Therefore, future studies could evaluate how time to TPE interacts with other factors to predict outcomes.

This study also found the association between time to TPE and outcomes to be non-linear. This non-linear association may suggest diversity in TTP clinical presentations. Of 66 patients with TTP on the Oklahoma TTP Registry, clinical diversity was illustrated in ten patients as follows: 1) prolonged prodrome of mild symptoms, 2) sudden onset of critical illness with multi-organ dysfunction, 3) stroke without hematologic manifestations, and 4) association with other life-threatening diseases (such as infections and systemic lupus erythematosus) [27]. TTP may also present with asymptomatic thrombocytopenia or small bowel ischemia [28,29]. The diversity of presentation may suggest that, compared to patients with mild symptoms at diagnosis, patients who are acutely ill at presentation may require more urgent treatment initiation. Nevertheless, how distinct clinical presentations impact TTP outcomes remains poorly understood. Understanding the relationship between clinical presentations, patterns and outcomes may guide optimal management strategies for specific subpopulations. Nevertheless, it is important to note that the cohort, which is focused on patients with suspected TTP, may also include patients with other TMAs [30]. Therefore, studies to evaluate the non-linear relationship of time to TPE with outcomes are needed in a cohort of patients with confirmed TTP.

The primary strength of this study is the use of a robust and rigorous methodology enhanced by combining a nonparametric approach with bootstrapping and clustering-based analysis. Additional strengths include the use of public use data files from REDS-III – a multicenter database optimized to provide accurate data regarding plasma issue. Nevertheless, REDS-III data is dated. Therefore, it may not account for current trends in TTP diagnosis and management. With the 2019 Food and Drug Administration (FDA) approval and evolving use of caplacizumab, the effect of time to TPE initiation on outcomes may have changed. Additionally, although this study uses a validated strategy to identify patients with suspected TTP, the database lacks key diagnostic variables to identify patients with true TTP including ADAMTS13 activity and variables to calculate the PLASMIC score (mean corpuscular volume) or French score (anti-neutrophil antibodies) [22,26,31]. Without these data, these findings can only be applied to a broad unselected population of patients with TMAs in whom TPE has been initiated. It cannot be generalized to patients with confirmed TTP [12]. Also, to avoid reverse causation bias from outcomes that may occur prior to TTP diagnosis, future studies could evaluate the association between time to TPE and TTP-specific outcomes such as platelet recovery, refractoriness, exacerbation, and relapse. Notwithstanding these limitations, this study is an important milestone in understanding the association between time to TPE initiation and outcomes.

## Conclusion

In patients with suspected TTP, the threshold for time to TPE initiation may be greater than recommended by the guidelines. Nevertheless, when the non-linear association between time to TPE and outcomes and its low predictive capacity is considered, other factors besides time to TPE alone may be important in predicting outcomes. Future studies may help evaluate how time to TPE interacts with other factors to predict clinical outcomes.

## Conflicts of interest

Oluwatoyosi A.Onwuemene has received honoraria from and has served on an Advisory Board for Sanofi. This relationship is unrelated to the content presented in this manuscript.

## Appendix Supplementary materials

Image, application 1

Image, image 2
