# Supplementary material for: In patients with suspected thrombotic thrombocytopenic purpura, what is the optimal time to therapeutic plasma exchange?
Source: Hematol Transfus Cell Ther. 2025 Dec 12;48(1):106223. doi: 10.1016/j.htct.2025.106223 (PMC12757464; doi:10.1016/j.htct.2025.106223)
Supplement: Supplementary file 1 [file mmc1.docx]

**SUPPLEMENTARY DATA**

**Supplementary methods – Sensitivity Analysis**

1. ***Parametric Model***

The parametric model was used for the following cohorts 1) patients with suspected thrombotic thrombocytopenic purpura (TTP), 2) patients who underwent ≥2 therapeutic plasma exchange (TPE) procedures, and 3) after removing potential outliers (time to TPE >5 days). This methodology was also used based on the cluster analysis for the following cohorts 1) after removing outlier clusters, and 2) after removing clusters with time to TPE 100-200 h. For all parametric models, we first checked the assumption of linearity between time to TPE and log odds of the primary composite outcome. Given the non-linear association identified for all cohorts, we visually observed potential knot placements and utilized cubic splines in the model formulation [1]. We then performed logistic regression to determine the optimal threshold for TPE initiation.

1. **Nonparametric Model**

Given the non-linear association identified between time to TPE and log odds of the primary outcome, we also evaluated generalized additive modeling (GAM). GAM extends traditional logistic regression by allowing for flexible, non-linear relationships between the predictor of interest (time to treatment) and the outcome. Thus, we avoided the need to select knots and the subsequent use of splines. This methodology was used in our cohort of patients with suspected TTP. Finally, we used the nonparametric model with bootstrapping (‘cutpointr”’ package in R) for the cluster analysis after excluding clusters with time to TPE of 100-200 h [2].

**Supplementary results – bootstrapping**

The bootstrapping results (1000 resamples) identified a median time threshold for TPE initiation of 14.1 h (interquartile range [IQR] 13.5 – 25.4 h, see Supplementary Figure 1).

**Supplementary results – clustering analysis**

| **Supplementary Table 1. Clustering based on time to TPE initiation** | | | | | | | |
| --- | --- | --- | --- | --- | --- | --- | --- |
| **Variable** | **Cluster #1**  **(n = 74)** | **Cluster #2**  **(n = 33)** | **Cluster #3**  **(n = 21)** | **Cluster #4**  **(n = 11)** | **Cluster #5**  **(n = 6)** | **Cluster #6**  **(n = 3)** | **Suspected TTP**  **(n = 148)** |
| **Time to TPE Initiation** | | | | | | | |
| **Time to TPE (h)** |  |  |  |  |  |  |  |
| Mean (SD) | 9.3 (5.6) | 37.6 (10.3) | 90.6 (21.6) | 194.4 (24.5) | 310.0 (46.6) | 534.7 (77.0) | 63.8 (101.6) |
| Median | 7.4 | 40.2 | 88.9 | 195.7 | 304.3 | 560.5 | 22.6 |
| Range | (0.6-21.6) | (23.5-53.7) | (66.1-131.8) | (162.9-234.3) | (261.2-375.6) | (448.2-595.5) | (0.6-595.5) |
| **Demographics** | | | | | | | |
| **Age (years)** |  |  |  |  |  |  |  |
| Mean (SD) | 48.1 (16.6) | 54.1 (18.4) | 51.5 (22.7) | 43.7 (21.1) | 55.8 (15.8) | 61.3 (12.5) | 50.2 (18.2) |
| Median | 47.5 | 51.0 | 51.0 | 41.0 | 57.5 | 61.0 | 50.0 |
| Range | (18.0-82.0) | (12.0-85.0) | (13.0-87.0) | (7.0-76.0) | (35.0-73.0) | (49.0-74.0) | (7.0-87.0) |
| **Gender** |  |  |  |  |  |  |  |
| Female | 56 (75.7%) | 23 (69.7%) | 13 (61.9%) | 8 (72.7%) | 3 (50.0%) | 1 (33.3%) | 104 (70.3%) |
| Male | 18 (24.3%) | 10 (30.3%) | 8 (38.1%) | 3 (27.3%) | 3 (50.0%) | 2 (66.7%) | 44 (29.7%) |
| Race |  |  |  |  |  |  |  |
| White | 43 (58.1%) | 19 (57.6%) | 17 (81.0%) | 8 (72.7%) | 6 (100.0%) | 3 (100.0%) | 96 (64.9%) |
| Black or Afro-American | 22 (29.7%) | 7 (21.2%) | 2 (9.5%) | 1 (9.1%) | 0 (0.0%) | 0 (0.0%) | 32 (21.6%) |
| Asian | 1 (1.4%) | 2 (6.1%) | 0 (0.0%) | 0 (0.0%) | 0 (0.0%) | 0 (0.0%) | 3 (2.0%) |
| Other | 2 (2.7%) | 3 (9.1%) | 1 (4.8%) | 1 (9.1%) | 0 (0.0%) | 0 (0.0%) | 7 (4.7%) |
| Not Reported | 6 (8.1%) | 2 (6.1%) | 1 (4.8%) | 1 (9.1%) | 0 (0.0%) | 0 (0.0%) | 10 (6.8%) |
| **Ethnicity** |  |  |  |  |  |  |  |
| Hispanic | 2 (2.7%) | 2 (6.1%) | 1 (4.8%) | 1 (9.1%) | 1 (16.7%) | 0 (0.0%) | 7 (4.7%) |
| Non-Hispanic | 66 (89.2%) | 29 (87.9%) | 19 (90.5%) | 9 (81.8%) | 5 (83.3%) | 3 (100.0%) | 131 (88.5%) |
| Not Specified | 6 (8.1%) | 2 (6.1%) | 1 (4.8%) | 1 (9.1%) | 0 (0.0%) | 0 (0.0%) | 10 (6.8%) |
| **Comorbidities** | | | | | | | |
| **Diabetes** | 17 (23.0%) | 7 (21.2%) | 5 (23.8%) | 1 (9.1%) | 0 (0.0%) | 3 (100.0%) | 33 (22.3%) |
| **Heart Failure** | 5 (6.8%) | 4 (12.1%) | 4 (19.0%) | 3 (27.3%) | 0 (0.0%) | 2 (66.7%) | 18 (12.2%) |
| **Renal Disease** | 39 (52.7%) | 26 (78.8%) | 16 (76.2%) | 9 (81.8%) | 5 (83.3%) | 3 (100.0%) | 98 (66.2%) |
| **Hepatic Disease** | 4 (5.4%) | 3 (9.1%) | 3 (14.3%) | 4 (36.4%) | 1 (16.7%) | 2 (66.7%) | 17 (11.5%) |
| **Stroke** | 6 (8.1%) | 6 (18.2%) | 4 (19.0%) | 2 (18.2%) | 0 (0.0%) | 1 (33.3%) | 19 (12.8%) |
| **Transient Ischemic Attack** | 0 (0.0%) | 0 (0.0%) | 2 (9.5%) | 0 (0.0%) | 0 (0.0%) | 0 (0.0%) | 2 (1.4%) |
| **Venous Thrombosis** | 6 (8.1%) | 10 (30.3%) | 2 (9.5%) | 2 (18.2%) | 3 (50.0%) | 2 (66.7%) | 25 (16.9%) |
| **Pulmonary Embolism** | 4 (5.4%) | 1 (3.0%) | 1 (4.8%) | 0 (0.0%) | 1 (16.7%) | 0 (0.0%) | 7 (4.7%) |
| **Immune Thrombocytopenia** | 8 (10.8%) | 1 (3.0%) | 2 (9.5%) | 0 (0.0%) | 0 (0.0%) | 0 (0.0%) | 11 (7.4%) |
| **Evans Syndrome** | 0 (0.0%) | 0 (0.0%) | 1 (4.8%) | 0 (0.0%) | 0 (0.0%) | 0 (0.0%) | 1 (0.7%) |
| **SLE** | 2 (2.7%) | 1 (3.0%) | 1 (4.8%) | 1 (9.1%) | 0 (0.0%) | 1 (33.3%) | 6 (4.1%) |
| **APS** | 4 (5.4%) | 2 (6.1%) | 3 (14.3%) | 1 (9.1%) | 0 (0.0%) | 1 (33.3%) | 11 (7.4%) |
| **Admission Labs** | | | | | | | |
| **Hemoglobin** |  |  |  |  |  |  |  |
| Mean (SD) | 9.4 (2.2) | 9.8 (2.5) | 10.0 (2.1) | 9.9 (2.2) | 9.3 (1.2) | 12.0 (3.1) | 9.7 (2.3) |
| Median | 9.3 | 9.3 | 10.0 | 10.2 | 8.8 | 12.4 | 9.4 |
| Range | (4.7-16.4) | (4.8-15.5) | (6.4-14.0) | (7.1-15.3) | (7.9-11.3) | (8.8-14.9) | (4.7-16.4) |
| **Platelets** |  |  |  |  |  |  |  |
| Mean (SD) | 36.7 (56.8) | 70.5 (85.5) | 108.5 (87.6) | 120.1 (86.1) | 174.7 (160.0) | 162.7 (71.9) | 68.8 (85.2) |
| Median | 19.0 | 42.5 | 82.0 | 119.0 | 113.0 | 171.0 | 36.0 |
| Range | (4.0-333.0) | (5.0-380.0) | (10.0-305.0) | (13.0-273.0) | (34.0-464.0) | (87.0-230.0) | (4.0-464.0) |
| **Lactate Dehydrogenase** |  |  |  |  |  |  |  |
| Mean (SD) | 1194.6 (951.2) | 1043.6 (950.4) | 792.3 (641.7) | 1189.1 (952.0) | 601.8 (383.2) | 343.7 (57.0) | 1062.3 (898.7) |
| Median | 846.5 | 700.5 | 610.0 | 577.0 | 533.0 | 372.0 | 730.0 |
| Range | (207.0-5002.0) | (126.0-3797.0) | (161.0-2864.0) | (307.0-2797.0) | (235.0-1235.0) | (278.0-381.0) | (126.0-5002.0) |
| **Creatinine** |  |  |  |  |  |  |  |
| Mean (SD) | 2.0 (2.1) | 2.2 (1.2) | 1.9 (1.4) | 2.8 (2.5) | 1.8 (1.2) | 1.8 (1.2) | 2.1 (1.8) |
| Median | 1.2 | 2.0 | 1.2 | 1.3 | 1.5 | 1.2 | 1.4 |
| Range | (0.6-14.2) | (0.5-5.6) | (0.7-5.7) | (0.5-7.3) | (0.8-4.2) | (1.0-3.1) | (0.5-14.2) |
| **Outcomes** | | | | | | | |
| **Composite outcome** | 38 (51.4%) | 24 (72.7%) | 14 (66.7%) | 6 (54.5%) | 5 (83.3%) | 2 (66.7%) | 89 (60.1%) |
| **Time to platelet recovery (d)** |  |  |  |  |  |  |  |
| Mean (SD) | 5.6 (4.0) | 11.5 (13.2) | 11.1 (10.5) | 7.1 (1.3) | 16.9 (N/A) | N/A | 7.6 (8.0) |
| Median | 4.6 | 4.6 | 7.1 | 7.1 | 16.9 | N/A | 4.6 |
| Range | (0.3-26.3) | (1.1-39.5) | (2.9-32.9) | (6.2-8.0) | (16.9-16.9) | N/A | (0.3-39.5) |
| **Refractory TTP** | 9 (18.0%) | 6 (35.3%) | 5 (41.7%) | 2 (28.6%) | 2 (100.0%) | 1 (100.0%) | 25 (28.1%) |
| **TTP relapse** | 6 (8.1%) | 2 (6.1%) | 0 (0.0%) | 0 (0.0%) | 0 (0.0%) | 0 (0.0%) | 8 (5.4%) |
| **TTP exacerbation** | 13 (17.6%) | 2 (6.1%) | 3 (14.3%) | 0 (0.0%) | 1 (16.7%) | 0 (0.0%) | 19 (12.8%) |
| APS: antiphospholipid syndrome; SD: Standard deviation; SLE: Systemic Lupus Erythematosus; N/A: Not applicable; TTP: Thrombotic thrombocytopenic purpura | | | | | | | |

**Cluster #1: “Prompt TPE initiation with the best outcomes”**

This cluster consisted of younger (48.1 ± 16 years old) and predominantly female (75.7%) patients. This cluster had the shortest mean time to TPE initiation (9.3 ± 5.6 h). Additionally, this cluster had the best rates of the primary composite outcome (51.4%). Finally, this cluster had the shortest time to platelet recovery (5.6 ± 4.0 days) and lowest rate of refractory TTP (18.0%).

**Cluster #2: “Rapid TPE initiation with worse outcomes”**

This cluster consisted of older (54.1 ± 18.4 years old) and predominantly female (69.7%) patients. This cluster had the second shortest mean time to TPE initiation (37.6 ± 10.3 h). Despite rapid TPE initiation, patients from this cluster had the second highest rate of the composite outcome (72.7%). Additionally, these patients had second longest mean time to platelet recovery (11.5 ± 13.2 days). Finally, refractory TTP occurred in 35.3% of the patients.

**Cluster #3: “Small delay in TPE initiation with worse outcomes”**

This cluster consisted of older (51.5 ± 22.7 years old) and predominantly female (61.9%) patients. Mean time to TPE initiation was 90.6 ± 21.6 h, which is higher than the average for the overall cohort. Patients from this cluster had worse outcomes: composite outcome (66.7%), time to platelet recovery (11.1 ± 10.5 days) and refractory TTP (41.7%).

**Cluster #4 “Long delay in TPE initiation with better outcomes”**

This cluster consisted of younger (43.7 ± 21.1 years old) and predominantly female (72.7%) patients. Mean time to TPE was 194.4 ± 24.5 h. Despite the long delay for TPE initiation, patients from this cohort had the second lowest rate of the composite outcome (54.5%) and refractory TTP (28.6%). Additionally, time to platelet recovery was 7.1 ± 1.3 days (the second shortest mean time).

**Cluster #5: “Huge delay in TPE initiation with the worst outcomes”**

This cluster consisted of older patients (55.8 ± 15.8 years old) and had 50% of males. A huge delay in TPE initiation occurred in this cluster – mean time was 310.0 ± 46.6 h. This cluster had the worst outcomes rates: composite outcome (83.3%) and refractory TTP (100%). Additionally, this cluster had the longest time to platelet recovery (16.9 days).

**Cluster #6: “Extreme delay in TPE initiation with worse outcomes”**

This cluster consisted of older (61.3 ± 12.5 years old) and predominantly male (66.7%) patients. This cluster had the longest mean time to TPE initiation (534.7 ± 77.0 h). Despite being the cluster with the longest time to TPE initiation, the composite outcome occurred in only 66.7% of patients, which is better than cluster #2 and #5. Due to the low number of patients and missing lab data, mean time to platelet recovery was not available. Refractory TTP occurred in 100% of the patients.

**References**

[1] Gauthier J, Wu QV, Gooley TA. Cubic splines to model relationships between continuous variables and outcomes: a guide for clinicians. Bone Marrow Transplant 2020;55:675–80. https://doi.org/10.1038/s41409-019-0679-x.

[2] Thiele C, Hirschfeld G. **cutpointr**: Improved Estimation and Validation of Optimal Cutpoints in *R*. J Stat Soft 2021;98. https://doi.org/10.18637/jss.v098.i11.

**Figure Legend**

**Supplementary Figure 1:** Optimal time to therapeutic plasma exchange threshold identified by bootstrapping (1000 resamples). Median time to therapeutic plasma exchange threshold identified of 14.1 h (IQR 13.5 – 25.4 h)


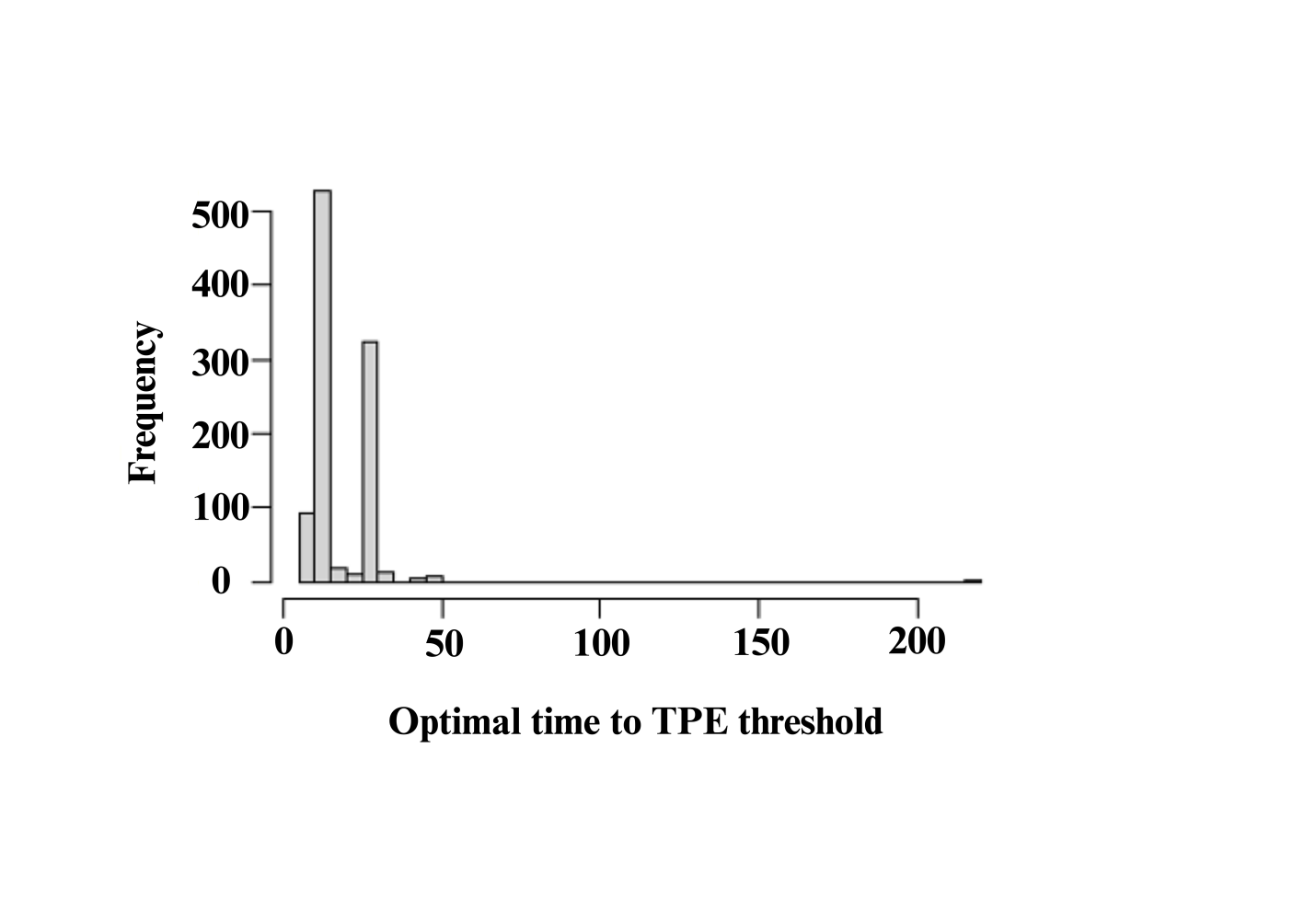


TPE: Therapeutic plasma exchange.

**Appendix**

| **Table 1. ICD and CPT Codes** | | | |
| --- | --- | --- | --- |
| **Variables** | **ICD-9 Codes** | **ICD-10 Codes** | **CPT Code** |
| **Therapeutic Plasma Exchange** | 99.71 | 6A550Z3, 6A551Z3 | 36514 |
| **Bleeding** | | | |
| **Gastrointestinal Bleeding** | 455.8, 456.0, 456.20 456.8, 530.21, 530.7, 530.82, 531.0x, 531.2x, 531.4x, 531.6x, 532.0x, 532.2x, 532.4x, 532.6x, 533.0x, 533.2x, 533.4x, 533.6x, 534.0x, 534.2x, 534.4x, 534.6x, 535.01, 535.11, 535.21, 535.31, 535.41, 535.51, 535.61, 535.71, 537.83, 537.84, 562.02, 562.03, 562.12, 562.13, 569.3, 569.85, 577.8, 578.x, 784.8, 569.86 | I85.01, I85.11, K20.81, K22.11, K25.2, K25.4, K25.6, K26.0, K26.2, K26.4, K26.6, K27.0, K27.2, K27.4, K27.6, K28.0, K28.2, K28.4, K28.6, K29.01, K29.21, K29.31, K29.41, K29.51, K29.61, K29.71, K29.81, K29.91, K31.811, K31.82, K50.011, K50.111, K50.811, K50.911, K51.011, K51.211, K51.311, K51.411, K51.511, K51.811, K51.911, K55.21, K57.01, K57.11, K57.13, K57.21, K57.31, K57.33, K57.41, K57.51, K57.53, K57.81, K57.91, K57.93, K62.5, K92.0, K92.1, K92.2, D78.21, D78.22, K22.6, K25.0, K20.91, K21.01, K91.840, K91.841 | 44.43 |
| **Intracranial Haemorrhage** | 430, 431, 432.0, 432.1, 432.9, 852.0x, 852.2x, 852.4x, 853.0x, 853.1x | I60, I60.0, I60.00, I60.01, I60.02, I60.1, I60.10, I60.11, I60.12, I60.2, I60.3, I60.30, I60.31, I60.32, I60.4, I60.5, I60.50, I60.51, I60.52, I60.6, I60.7, I60.8, I60.9, I61, I61.0, I61.1, I61.2, I61.3, I61.4, I61.5, I61.6, I61.8, I61.9, I62, I62.0, I62.00, I62.01, I62.02, I62.03, I62.1, I62.9, I60.20, I60.21, I60.22, S06.4X0A, S06.4X1A, S06.4X2A, S06.4X3A, S06.4X4A, S06.4X5A, S06.4X6A, S06.4X7A, S06.4X8A, S06.4X9A, G97.51, G97.52 | - |
| **Genitourinary Haemorrhage** | 580.9, 596.7, 596.8, 596.7x, 599.7x 602.1x, 620.1, 621.4, 626.2, 626.5, 626.7, 626.8, 626.9 | R31.0, R31.9, N42.1, N93.8, N93.9, N95.0, N99.820, N99.821 | - |
| **Pulmonary Haemorrhage** | 786.3x | R04.2, R04.89, R04.9, J95.830, J95.831 | - |
| **Other Haemorrhage** | 285.1, 360.43, 362.43, 362.81, 363.61, 363.62, 363.72, 364.41, 372.72, 374.81, 376.32, 377.42, 379.23, 423.0x, 459.0, 568.81, 719.1x, 784.7, 784.8, 958.2, 997.02, 998.11, 729.92 | H05.23, H05.231, H05.232, H05.233, H21.0, H21.00, H21.01, H21.02, H21.03, H31.3, H31.30, H31.301, H31.302, H31.303, H31.309, H31.31, H31.311, H31.312, H31.313, H31.319, H31.31, H31.311, H31.312, H31.313, H31.319, H31.41, H31.411, H31.412, H31.413, H31.419, H35.6, H35.60, H35.61, H35.62, H35.63, H35.73, H35.731, H35.732, H35.733, H35.739, H43.1, H43.10, H43.11, H43.12, H43.13, H44.81, H44.811, H44.812, H44.813, H44.819, H47.02, H47.021, H47.022, H47.023, H47.029, H61.12, H61.121, H61.122, H61.123, H61.129, I31.2, K66.1, M25.00, M25.01, M25.011, M25.012, M25.019, M25.02, M25.021, M25.022, M25.029, M25.03, M25.031, M25.032, M25.039, M25.04, M25.041, M25.042, M25.049, M25.05, M25.051, M25.052, M25.059, M25.06, M25.061, M25.062, M25.069, M25.07, M25.071, M25.072, M25.073, M25.074, M25.075, M25.076, M25.08, M79.81, R04.0, R04.1, R23.3, R58, H05.239, H59.311, H59.312, H59.313, H59.319, H59.321, H59.322, H59.323, H59.329, H95.41, H95.42, I97.610, I97.611, I97.618, I97.62, L76.21, L76.22, M96.830, M96.831 | 99.04 |
| **Thrombosis** | | | |
| **Arterial thrombosis** | **Myocardial Infarction**  410.0, 410.00, 410.01, 410.02, 410.10, 410.11, 410.12, 410.20, 410.21, 410.22, 410.30, 410.31, 410.32, 410.40, 410.41, 410.42, 410.50, 410.51, 410.52, 410.60, 410.61, 410.62, 410.70, 410.71, 410.72, 410.80, 410.81, 410.82, 410.9, 410.91, 410.92, 410.1, 410.2, 410.3, 410.4, 410.5, 410.6, 410.7, 410.8, 410.9, 411.81, 411.0  **Ischemic Stroke**  433.01, 433.11, 433.21, 433.31, 433.81, 433.91, 434.01, 434.1, 434.10, 434.11, 434.90, 434.91, 437.1  **Amputation // Extremities**  444.2, 444.21, 444.22, 445.01, 445.02, 84.0, 84.00, 84.01, 84.02, 84.03, 84.04, 84.05, 84.06, 84.07, 84.08, 84.09, 84.1, 84.10, 84.11, 84.12, 84.13, 84.14, 84.15, 84.16, 84.17, 84.18, 84.19,  **Others**  444, 444.0, 444.01, 444.09, 444.1, 444.8, 444.81, 444.89, 444.9, 557.0, 557.1, 557.9, 445.81, 445.89 | **Myocardial Infarction**  I21.xx, I22.xx, I23.xx, I24.9.  **Ischemic Stroke**  I63.xx, G46.xx.  **Amputation // Extremities**  0Y6H0Z3, 0Y6J0Z3, 0Y6H0Z1, 0Y6H0Z2, 0Y6H0Z3, 0Y6J0Z1, 0Y6J0Z2, 0Y6J0Z3, 0Y6F0ZZ, 0Y6G0ZZ, 0Y6C0Z1, 0Y6C0Z2, 0Y6C0Z3, 0Y6D0Z1, 0Y6D0Z2, 0Y6D0Z3, 0Y670ZZ, 0Y680ZZ, 0Y620ZZ, 0Y630ZZ, 0Y640ZZ, 0Y6M0Z0, 0Y6N0Z0, 0Y6P0Zx, 0Y6Q0Zx, 0Y6R0Zx, 0Y6S0Zx, 0Y6U0Zx, "0Y6V0Zx, 0Y6W0Zx, 0Y6X0Zx, 0Y6Y0Zx, 0Y6M0Zx, 0Y6N0Zx, 0Y6T0Zx, I74.2, I74.3, I74.4.  **Others**  I74.0, I74.01, I74.09, I74.1, I74.10, I74.11, I74.19, I74.5, I74.8, I74.9, K55.01, K55.011, K55.012, K55.019, K55.02, K55.021, K55.022, K55.029, K55.03, K55.031, K55.032, K55.039, K55.04, K55.041, K55.042, K55.049, K55.05, K55.051, K55.052, K55.059, K55.06, K55.061, K55.062, K55.069, H34.0, H34.00, H34.01, H34.02, H34.03, H34.1, H34.10, H34.11, H34.12, H34.13, H34.2, H34.21, H34.211, H34.212, H34.213, H34.219, H34.23, H34.231, H34.232, H34.233, H34.239 | CPT-27290, CPT-27590, CPT-27591, CPT-27592, CPT-27594, CPT-27596, CPT-27880, CPT-27881, CPT-27882, CPT-27884, CPT-27886, CPT-27888, CPT-28800, CPT-28805, CPT-28810, CPT-28820, CPT-28825 |
| **Venous Thrombosis** | **Lower Extremities**  451.0, 451.11, 451.19, 451.2, 453.40, 453.41, 453.42, 453.50, 453.51, 453.52, 453.6.  **Upper Extremities**  451.82, 451.83, 451.84, 453.71, 453.72, 453.73, 453.81, 453.82, 453.83.  **Pulmonary Embolism**  415.1, 415.11, 415.13, 415.19  **Other VTE**  451.81, 451.89, 451.9, 452, 453.0, 453.1, 453.2, 453.3, 453.74, 453.75, 453.76, 453.77, 453.79, 453.84, 453.85, 453.86, 453.87, 453.89, 453.9, 437.6, 325, 671.30, 671.31, 671.33, 671.40, 671.42, 671.44, 671.90, 671.91, 671.92, 671.93, 671.94, V12.51, V12.52 | **Lower Extremities**  I82.4, I82.40, I82.401, I82.402, I82.403, I82.409, I82.411, I82.412, I82.413, I82.419, I82.42, I82.421, I82.422, I82.423, I82.429, I82.43, I82.431, I82.432, I82.433, I82.439, I82.44, I82.441, I82.442, I82.443, I82.449, I82.45, I82.451, I82.452, I82.453, I82.459, I82.46, I82.461, I82.462, I82.463, I82.469, I82.49, I82.491, I82.492, I82.493, I82.499, I82.4Y, I82.4Y1, I82.4Y2, I82.4Y3, I82.4Y9, I82.4Z, I82.4Z1, I82.4Z2, I82.4Z3, I82.4Z9, I82.501, I82.502, I82.503, I82.509, I82.511, I82.512, I82.513, I82.519, I82.521, I82.522, I82.523, I82.529, I82.531, I82.532, I82.533, I82.539, I82.541, I82.542, I82.543, I82.549, I82.551, I82.552, I82.553, I82.559, I82.561, I82.562, I82.563, I82.569, I82.591, I82.592, I82.593, I82.599, I82.5Y1, I82.5Y2, I82.5Y3, I82.5Y9, I82.5Z1, I82.5Z2, I82.5Z3, I82.5Z9, I82.811, I82.812, I82.813, I82.819.  **Upper Extremities**  I82.6, I82.60, I82.601, I82.602, I82.603, I82.609, I82.61, I82.611, I82.612, I82.613, I82.619, I82.62, I82.621, I82.622, I82.623, I82.629, I82.7, I82.70, I82.701, I82.701, I82.702, I82.703, I82.709, I82.711, I82.712, I82.713, I82.719, I82.721, I82.722, I82.723, I82.729.  **Pulmonary Embolism**  I26, I26.0, I26.01, I26.02, I26.09, I26.9, I26.92, I26.93, I26.94, I26.99.  **Other VTE**  I81, I82.0, I82.1, I82.2, I82.21, I82.210, I82.21, I82.22, I82.220, I82.221, I82.29, I82.290, I82.291, I82.3, I82.A, I82.A1, I82.A11, I82.A12, I82.A13, I82.A19, I82.A21, I82.A22, I82.A23, I82.A29, I82.B, I82.B11, I82.B12, I82.B13, I82.B19, I82.B2, I82.B21, I82.B22, I82.B23, I82.B29, I82.C, I82.C1, I82.C11, I82.C12, I82.C13, I82.C19, I82.C2, I82.C21, I82.C22, I82.C23, I82.C29, I82.8, I82.89, I82.890, I82.891, I82.9, I82.90, I82.91 | - |
| **Comorbidities** | | | |
| **Diabetes** | 294.xx, 250.xx | E08.xxxx, E09.xxxx, E10.xxxx, E11.xxxx, E13.xxxx | - |
| **Heart Failure** | 398.91, 402.01, 402.11, 402.91, 404.01, 404.03, 404.11, 404.13, 404.91, 404.93, 428.xx | I09.81, I11.0, I13.0, I13.2, I50.xxx | - |
| **Renal Disease** | 580.0 580.4 580.81 580.89 580.9, 585.1, 581.0, 581.1, 581.2, 581.3, 581.81, 581.89, 581.9, 582.0, 582.1, 582.2, 582.4, 582.81, 582.89, 582.9, 583.0, 583.1, 583.2, 583.4, 583.6, 583.7, 583.81, 583.89, 583.9, 587, 584.5, 584.6, 584.7, 584.8, 584.9, 586, 585, 585.1, 585.2, 585.3, 585.4, 585.5, 585.6, 585.9, V42.0, V45.1, V45.11, V45.12, V56.0, V56.1, V56.2, V56.31, V56.32, V56.8, PR39.95, 403.01, 403.11, 403.91, 404.02, 404.03, 404.12, 404.13, 404.92, 404.93. | N00.xx, N01.xx, N02.xx, N03.xx, N04.xx, N05.xx, N06.xx, N07.xx, N08.xx, N11.xx, N12.xx, N14.xx, N16.xx, N13.721, N13.722, N13.729, N13.731, N13.732, N13.739, N17.xx, N18.xx, N19.xx, 5A1D70Z, 5A1D80Z, 5A1D90Z, 5A1D00Z, 5A1D60, 3E1M39Z | - |
| **Hepatic Disease** | 570, 571.0, 571.1, 571.2, 571.3, 571.40, 571.41, 571.42, 571.49, 571.5, 571.6, 571.8, 571.9, 572.0, 572.1, 572.2, 572.3, 572.4, 572.8, 573.0, 573.4, 573.5, 573.8, 573.9,789.1, V42.7, 070.0, 070.1, 070.2, 070.20, 070.21, 070.22, 070.23, 070.3, 070.31, 070.32, 070.33, 070.4, 070.41, 070.42, 070.43, 070.44, 070.49, 070.5, 070.51, 070.52, 070.53, 070.54, 070.59, 070.6, 070.70, 070.71, 070.9, 072.71, 571.41, 571.42, 571.49, 573.1, 573.2, 573.3,456.0, 456.1, 456.20, 456.21, 070.30 and 571.40 | K70.0, K70.10, K70.11, K70.2, K70.30, K70.31, K70.40, K70.41, K70.9, K71.0, K71.10, K71.11, K71.2, K71.3, K71.4, K71.50, K71.51, K71.6, K71.7, K71.8, K71.9, K72.00, K72.01, K72.10, K72.11, K72.90, K72.91, K73.0, K73.1, K73.2, K73.8, K73.9, K74.00, K74.01, K74.02, K74.1, K74.2, K74.3, K74.4, K74.5, K74.60, K74.69, K75.0, K75.2, K75.3, K75.4, K75.81, K75.89, K75.9, K76.0, K76.1, K76.2, K76.3, K76.4, K76.5, K76.6, K76.7, K76.81, K76.89, K76.89, K77, I85.00, I85.01, I85.10, I85.11 | - |
| **Stroke** | 433.01, 433.11, 433.21, 433.31, 433.81, 433.91, 434.01, 434.1, 434.10, 434.11, 434.90, 434.91, 437.1 | I63.xx, G46.xx. | - |
| **Transient Ischemic Attack** | 435.0, 435.1, 435.2, 435.3, 435.8, 435.9 | G45.0x, G45.1x, G45.2x, G45.3, G45.4x, G45.8x, G45.9x | - |
| **Antiphospholipid syndrome** | 289.81 | D68.61 | - |
| **Immune thrombocytopenia** | 287.31 | D69.3 | - |
| **Evans syndrome** | 287.32  **OR**  **BOTH** [287.31](http://www.icd9data.com/2014/Volume1/280-289/287/287.31.htm) + 283.0 | D69.41  **OR**  **BOTH** D69.3 + at least one of the following:  D59.0, D59.1, D59.10, D59.11, D59.12, D59.13, D59.19 | - |
| **Systemic lupus erythematosus** | 710.0 | M32.xx | - |
| **Thrombotic microangiopathy** | 446.6 | M31.1xx | - |
| ICD = International classification of disease codes; CPT = Current procedural terminology | | | |
