# Supplementary figures and images for: In patients with suspected thrombotic thrombocytopenic purpura, what is the optimal time to therapeutic plasma exchange?
Source: Hematol Transfus Cell Ther. 2025 Dec 12;48(1):106223. doi: 10.1016/j.htct.2025.106223 (PMC12757464; doi:10.1016/j.htct.2025.106223)

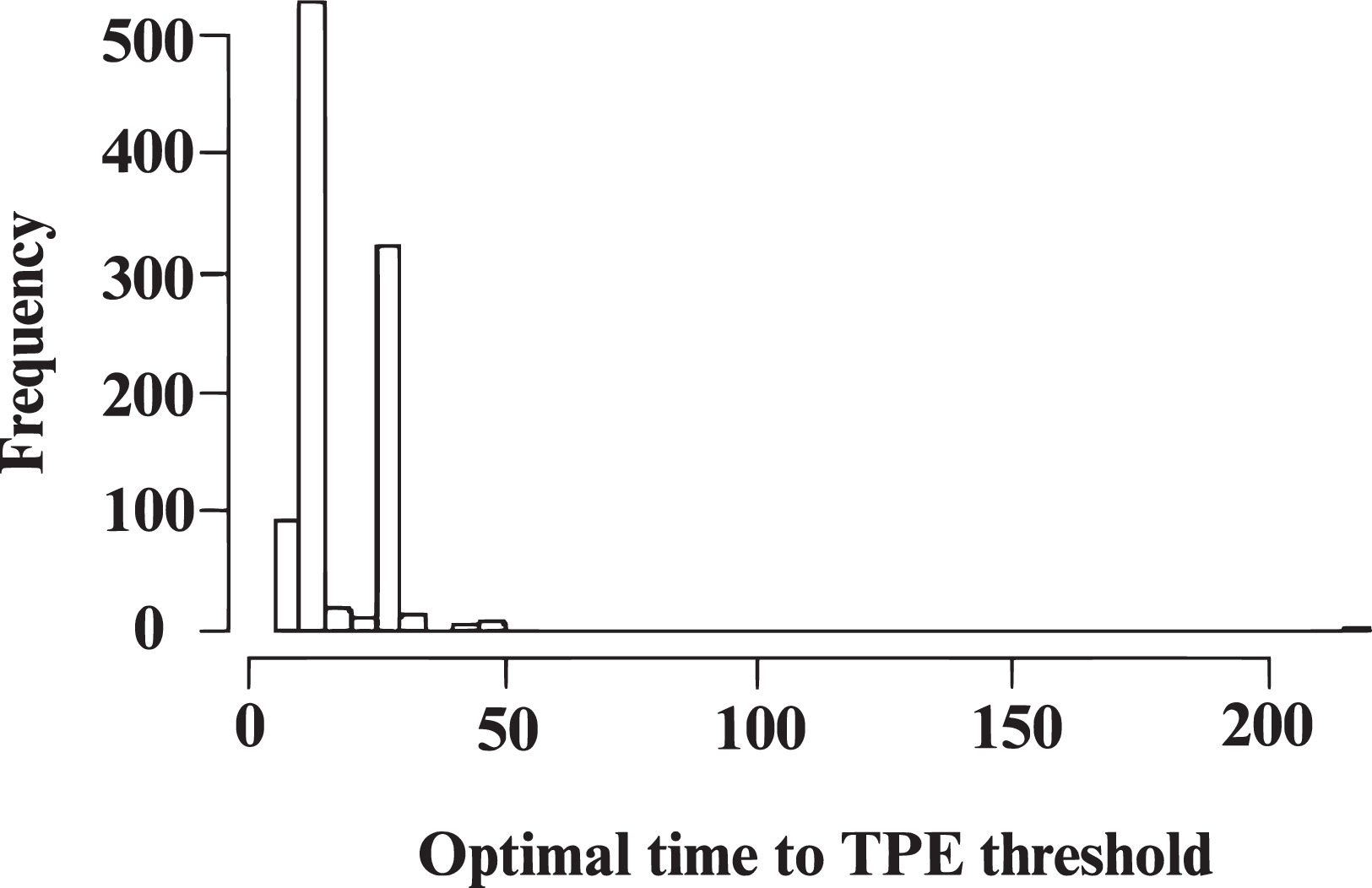

Supplement: Supplementary file 2 [file mmc2.jpg]
